# Fingertip dynamic response simulated across excitation points and frequencies

**DOI:** 10.1007/s10237-024-01844-4

**Published:** 2024-05-03

**Authors:** Gokhan Serhat, Katherine J. Kuchenbecker

**Affiliations:** 1 Department of Mechanical Engineering, KU Leuven, Spoorwegstr. 12, 8200 Bruges, Belgium; 2 Haptic Intelligence Department, Max Planck Institute for Intelligent Systems, Heisenbergstr. 3, 70569 Stuttgart, Germany

**Keywords:** Harmonic response, Human fingertip, Finite element analysis, Soft-tissue dynamics

## Abstract

Predicting how the fingertip will mechanically respond to different stimuli can help explain human haptic perception and enable improvements to actuation approaches such as ultrasonic mid-air haptics. This study addresses this goal using high-fidelity 3D finite element analyses. We compute the deformation profiles and amplitudes caused by harmonic forces applied in the normal direction at four locations: the center of the finger pad, the side of the finger, the tip of the finger, and the oblique midpoint of these three sites. The excitation frequency is swept from 2.5 to 260 Hz. The simulated frequency response functions (FRFs) obtained for displacement demonstrate that the relative magnitudes of the deformations elicited by stimulating at each of these four locations greatly depend on whether only the excitation point or the entire finger is considered. The point force that induces the smallest local deformation can even cause the largest overall deformation at certain frequency intervals. Above 225 Hz, oblique excitation produces larger mean displacement amplitudes than the other three forces due to excitation of multiple modes involving diagonal deformation. These simulation results give novel insights into the combined influence of excitation location and frequency on the fingertip dynamic response, potentially facilitating the design of future vibration feedback devices.

## Introduction

The fingertip plays a central role in the human sense of touch. To support the ubiquitous functions of tactile exploration, grasping, and manipulation, fingertips possess high concentrations of four distinct types of mechanoreceptors (Johansson and Vallbo 1979). Hence, these body parts exhibit a high sensitivity to both external static forces (i.e., pressure) and dynamic excitation (i.e., vibrations) (Löfvenberg and Johansson 1984).

The significance of the human fingertip has inspired continuous efforts devoted to understanding its dynamic characteristics. For instance, Pawluk and Howe (1999) investigated the dynamic response of the human finger pad during harmonic compression. Their findings showed that the accuracy of lumped response models is susceptible to small changes in the contact point. Later, Wiertlewski and Hayward (2012) measured the mechanical impedance of the fingertips by controlling the frequency and pressing force of a flat contact surface undergoing lateral vibrations. This study revealed that modeling the fingertip as a homogeneous solid would be a great over-simplification of the actual physics due to the sophisticated deformation behavior of the soft tissue. Similarly, Yildiz and Güçlü (2013) determined the complex moduli of the fingertips using oscillating indenters with different radii and positions. Their results indicated the major influence of the contactor size on the measured modulus and smaller effects of the indentation location.

The intricate anatomy and nonuniform properties of the tissue layers complicate the fingertip deformation mechanics and limit the usability of analytical models. Consequently, computational simulations have been prevalently employed to explore the dynamic behavior of fingertips. For example, Wu et al. (2007) created a 2D finite element (FE) model of the fingertip cross section to analyze its response to a vibrating contact surface. Their analyses showed that maximum soft-tissue strains can occur distant from the excitation source due to resonances. Later, a 3D FE model was developed by the same authors (Wu et al. 2009) and was used to investigate the dynamic deformations caused by cylindrical indenters with varied locations along the symmetry plane. Most recently, a high-fidelity 3D FE fingertip model named DigiTip was developed by the authors of the present study (Serhat and Kuchenbecker 2021). It was used to predict the free vibration modes up to 260 Hz and the forced vibration response for harmonic forces applied at the center of the finger pad. A version of this parametric model was customized to an individual and experimentally validated within another study by the authors (Gertler et al. 2022), where the simulated and measured frequency-dependent response amplitudes showed very close agreement with no parameter tuning.

Despite the high interest in this topic and the promising previous experimental and computational studies, a high-fidelity FE model has not yet been used to investigate *how excitation location and frequency influence the fingertip’s dynamic response*. This information can enable prediction of the fingertip response to different excitation sources as well as determination of the optimal stimulation locations for haptic rendering technology such as electromagnetic vibrotactile actuators (Choi and Kuchenbecker 2012; Gertler et al. 2022) and mid-air ultrasonic devices (Rakkolainen et al. 2021). In this paper, we focus on this gap and use finite element analysis (FEA) to compute the fingertip vibration responses to harmonic point forces acting normal to the skin. We apply the forces at four locations: the finger pad’s center, side, and tip, as well as the midpoint of these three spots on the skin’s surface. The excitation frequency of each force is systematically increased up to 260 Hz, which is around the maximum sensitivity frequency in the human hand (Gescheider et al. 2002). Our results reveal the significant difference between relative vibration amplitudes calculated at the excitation points themselves versus over the entire fingertip. In addition, excitation at the diagonal midpoint is found to provide the highest mean displacement amplitudes after about 225 Hz. These findings shed light on the combined influence of excitation location and frequency on the fingertip dynamic response.

## Materials and methods

### Finite element analysis

**Fig. 1:**
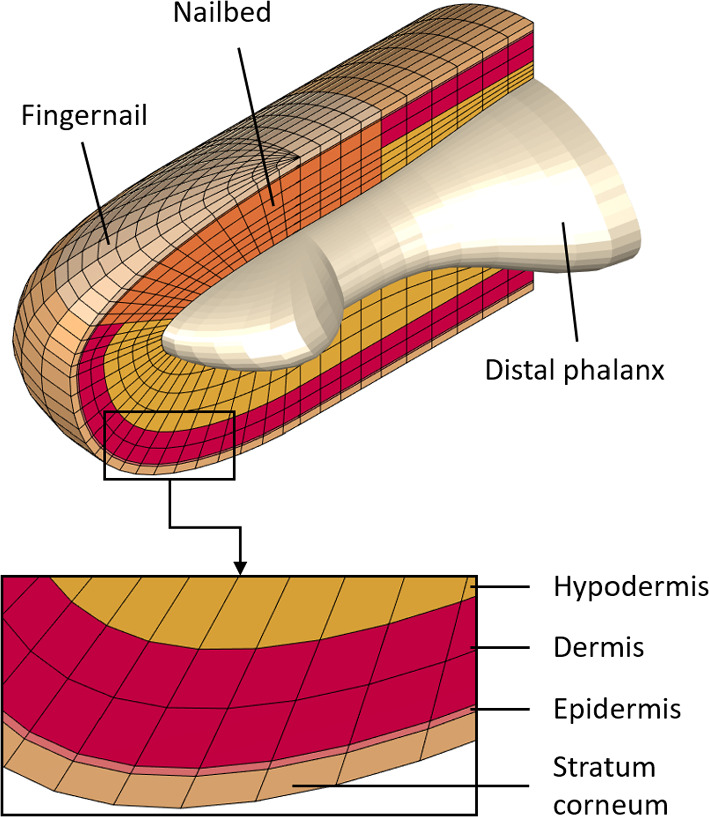
Section view of the 3D finite element model DigiTip, which consists of the stratum corneum, epidermis, dermis, hypodermis (subcutaneous tissue), distal phalanx (bone), nailbed, and fingernail

The calculations use our previously developed 3D high-fidelity finite element model called DigiTip (Serhat and Kuchenbecker 2021). That study can be referred to for all dimensions, material properties, and FEA parameters, along with the references used to determine all characteristics of the model. Figure 1 shows a section view of this FE model of the distal segment of a human fingertip; the full model of the tissue contains 11 637 nodes and 10 080 elements. The bone is assumed rigid, so the nailbed and hypodermis nodes in contact with the bone are immobilized. The other boundaries are free. The model does not include the fingerprints since they would not play a significant role in the tissue’s bulk dynamic deformation, while their modeling would require a very fine mesh, significantly increasing the computational cost.

The finite element model is first used for free vibration analysis. Denoting the global stiffness matrix by *K* and mass matrix by *M*, the natural frequencies ($\omega _n$) can be determined by solving the eigenvalue equation (Liu and Quek 2003):

1 $$\begin{aligned} \textrm{det}(K-{\omega _{n}}^2 M)=0 \end{aligned}$$

The mode shape vector ($X_n$) corresponding to each natural frequency can be obtained from the following relation:

2 $$\begin{aligned} (K-{\omega _{n}}^2 M)X_{n}=0 \end{aligned}$$

In the forced vibration analyses, damping is incorporated within the system by modifying the global stiffness to be complex with a loss factor $\eta$ as follows:

3 $$\begin{aligned} K^{*}=K(1+i\eta ) \end{aligned}$$

Then, for a given load vector *F* and excitation frequency $\omega$, the vector of nodal displacement amplitudes (*X*) can be found as

4 $$\begin{aligned} (K^{*}-\omega ^{2})X=F \end{aligned}$$

The response vector can also be approximated as the sum of several eigenvectors multiplied by their amplitudes $\xi _n$ (Harris and Piersol 2002):

5 $$\begin{aligned} X \approx X_{n}\xi _{n} \end{aligned}$$

Although we use the direct solution approach presented in Eq. (4) to ensure the accuracy of the resulting prediction, modal amplitudes $\xi _n$ can also be computed via the following equation:

6 $$\begin{aligned} (K^{*}-\omega ^{2}M)X_{n}\xi _{n}=F \end{aligned}$$

After computing the displacement FRFs, we use Eq. (6) to investigate the excitation level of the fingertip’s individual resonant modes. This analysis enables more precise identification of the modes contributing to the peak response amplitudes.

### Excitation

**Fig. 2:**
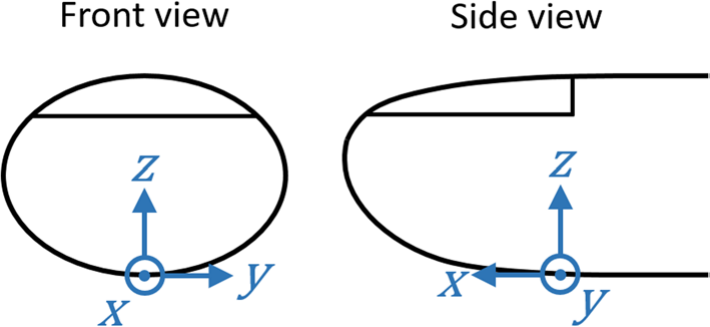
Front and side views of the fingertip showing the chosen Cartesian coordinate system

We define the positions of the forces applied on the outer skin through a Cartesian coordinate system. Figure 2 shows front and side views of the chosen origin and the coordinate axes relative to the fingertip.

**Fig. 3:**
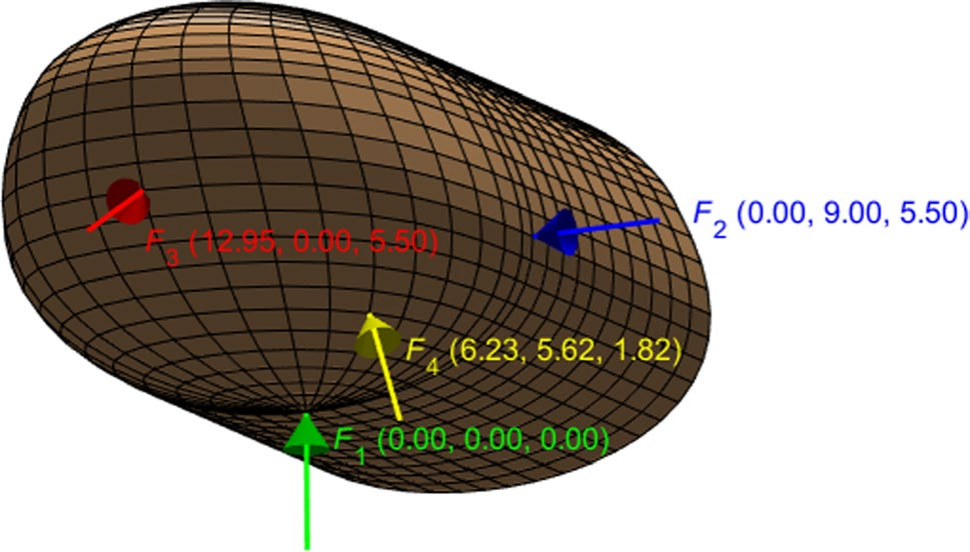
Four harmonic point forces exerted perpendicular to the surface of the fingertip. $F_1$, $F_2$, and $F_3$ are applied at the center of the finger pad (origin), the finger’s side, and the finger’s front, respectively. $F_4$ is approximately equidistant from the first three forces. The Cartesian coordinates of the excitation points are provided in millimeters in parentheses next to their respective forces

To investigate the influence of the excitation location on the dynamic response, we utilize the four different harmonic point forces shown in Fig. 3. $F_1$, $F_2$, and $F_3$ are applied at the center (origin), side, and front tip of the finger pad, respectively. $F_4$ is located approximately in the middle of these three forces. As depicted in the figure, all forces are perpendicular to the skin surface.

The excitation amplitude is set to be 0.002 N, which is approximately the weight of 0.2 g on Earth (0.2 gram-force), a moderate value for a typical mid-air haptic device (Raza et al. 2020). Only one excitation location is considered for each simulation. The forcing frequency is swept from 2.5 to 260 Hz with an increment of 2.5 Hz.

## Results and discussion

### Displacement amplitudes vs. excitation frequency

**Fig. 4:**
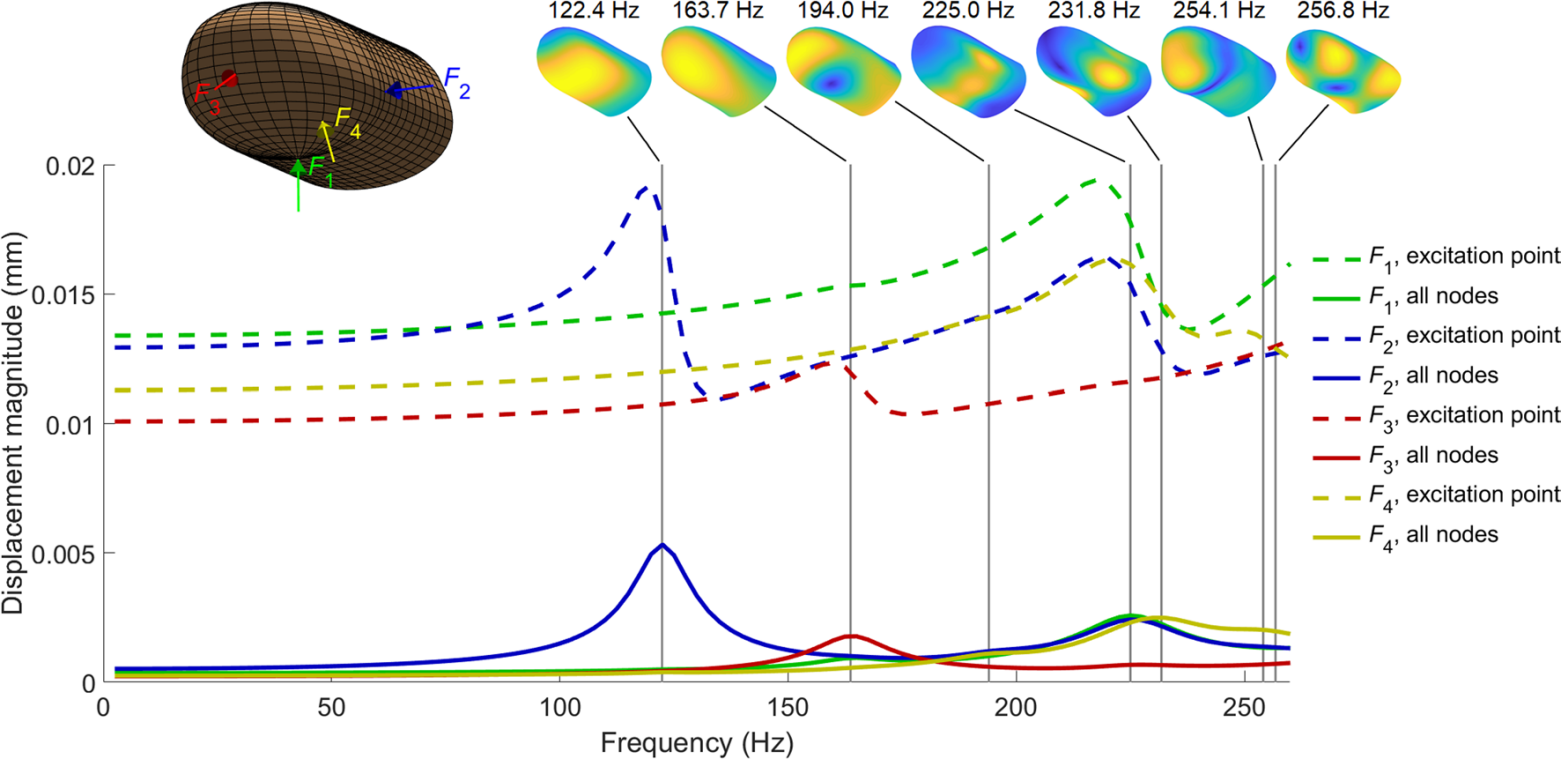
The displacement FRFs for the harmonic forces applied to four different points on the fingertip. The seven vibration modes present in the frequency range of interest are displayed at the top, where the yellow and dark blue regions correspond to the largest and smallest relative displacement magnitudes, respectively. The continuous curves at the bottom show the mean amplitudes across all model nodes, while the dashed curves at the top represent the responses at the excitation points

First, we calculate the displacement frequency response functions (FRFs) for the four harmonic point forces displayed in Fig. 3. Figure 4 shows the results for both the excitation point and the mean of all nodes in the model. Naturally, the mean amplitudes are smaller than the responses at the excitation point. To facilitate the interpretation of the FRFs, we also present the seven free vibration modes computed in our previous study for the 0–260 Hz range. Interested readers can better understand these vibration modes by watching the supplementary video associated with the original DigiTip paper (Serhat and Kuchenbecker 2021).

The displacement FRFs exhibit resonances around several modal frequencies. The first resonance observed at 122.4 Hz is associated with $F_2$, and it occurs due to the excitation of the first mode involving torsional deformation around the longitudinal axis (the distal-proximal direction parallel to the x-axis in Fig. 2) of the bone. The thinning of the distal phalanx beneath the finger pad facilitates the excitation of this mode since it reduces the bone support against lateral (in the radial-ulnar direction) loads. This first peak appears in both local and average displacement results due to the global rotational deformation of the entire fingertip.

The second resonance is evident only for $F_3$, which excites the second fingertip mode at 163.7 Hz. This mode also exhibits a global rotational motion, but around the lateral axis (the radial-ulnar direction parallel to the y-axis in Fig. 2). This behavior is reflected in the mean amplitudes, where $F_3$ induces the highest value at this frequency. The other three forces do not effectively excite the second mode; however, they still cause higher displacement amplitudes at the excitation point than on average across the model, indicating that their deformation profiles are mainly local. Hence, the modal dynamics of the fingertip may cause significant differences between relative vibration amplitudes calculated at the excitation points and over the entire fingertip.

The third mode at 194.0 Hz does not cause a significant resonance in any of the considered cases since it primarily comprises lateral motion around the distal end. This movement cannot be efficiently excited by any of the tested normal forces.

The next strong peaks in the mean displacement FRFs occur near 230 Hz due to the proximity of the fourth (225.0 Hz) and fifth (231.8 Hz) modes, which involve normal deformation of the finger pad center and the finger’s lower side regions (Serhat and Kuchenbecker 2021), respectively. These resonances are activated by all forces except $F_3$, which is far from the modal deformation sites. $F_1$ yields the highest local displacement amplitude because its position lies at the center of the moving area associated with the fourth mode and its direction is aligned with the modal motion.

When the excitation frequency is greater than 231.8 Hz, $F_4$ produces the largest mean displacement amplitude. This result stems from the excitation of multiple modes involving the deformation of diagonal regions. Note that $F_4$ does not cause the highest local or mean amplitude at the lower frequencies. Therefore, at frequencies around 250 Hz, applying an oblique dynamic force could be a good approach to increase the overall vibration amplitude within the fingertip.

### Modal amplitudes vs. excitation frequency

Next, we analyze modal amplitudes as a function of excitation frequency considering the first seven natural modes and four chosen excitation locations. This investigation breaks the overall deformation response into modes to facilitate quantification of the degree of excitation for each mode.

**Fig. 5:**
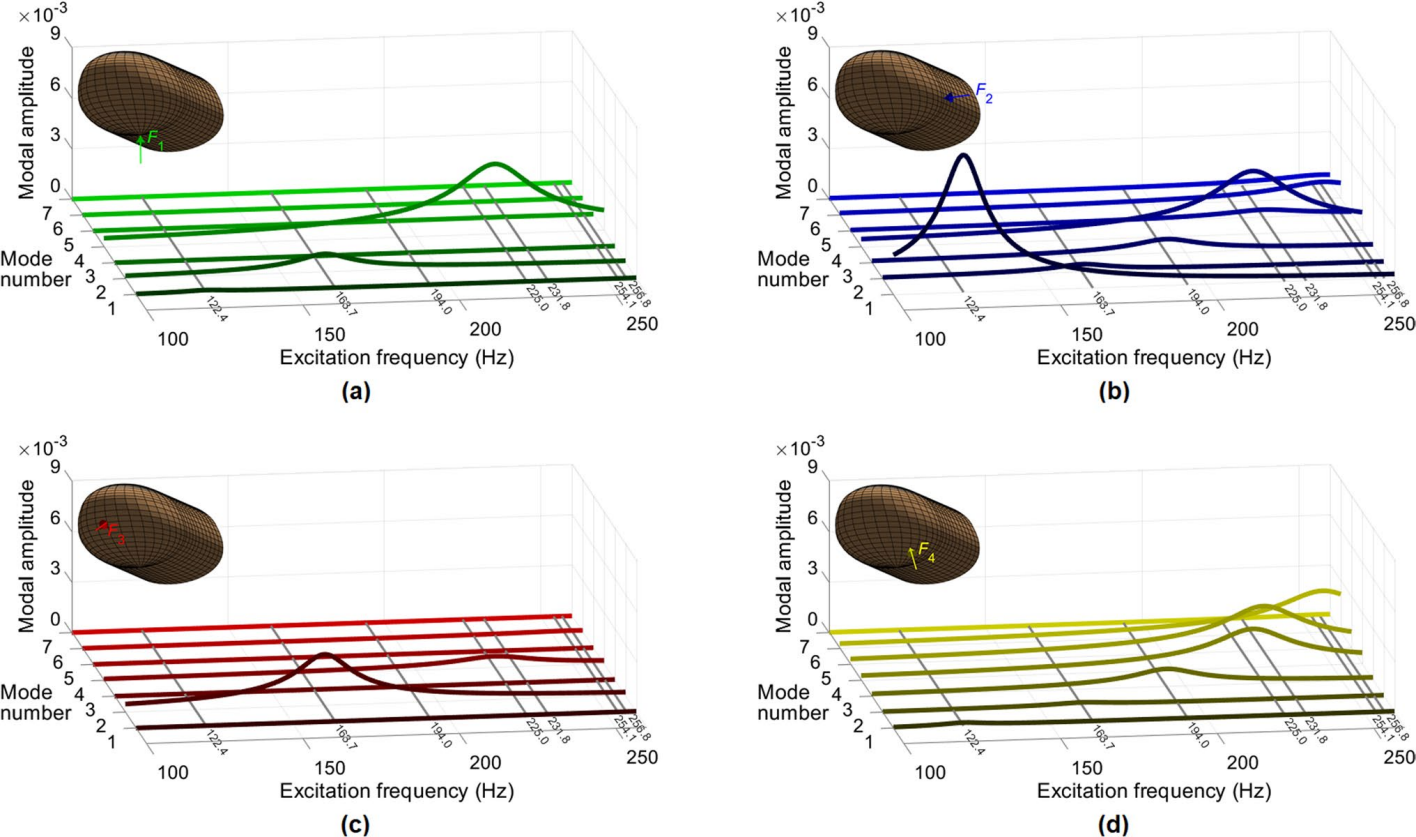
Amplitude vs. excitation frequency curves for the first seven natural modes and the four studied excitation locations

Figure 5a, b, c, and d shows the results for $F_1$, $F_2$, $F_3$, and $F_4,$ respectively. In the plots, the lower limit for the excitation frequency is selected as 100 Hz since the responses are not influenced by resonances below this frequency (Fig. 4).

**Fig. 6:**
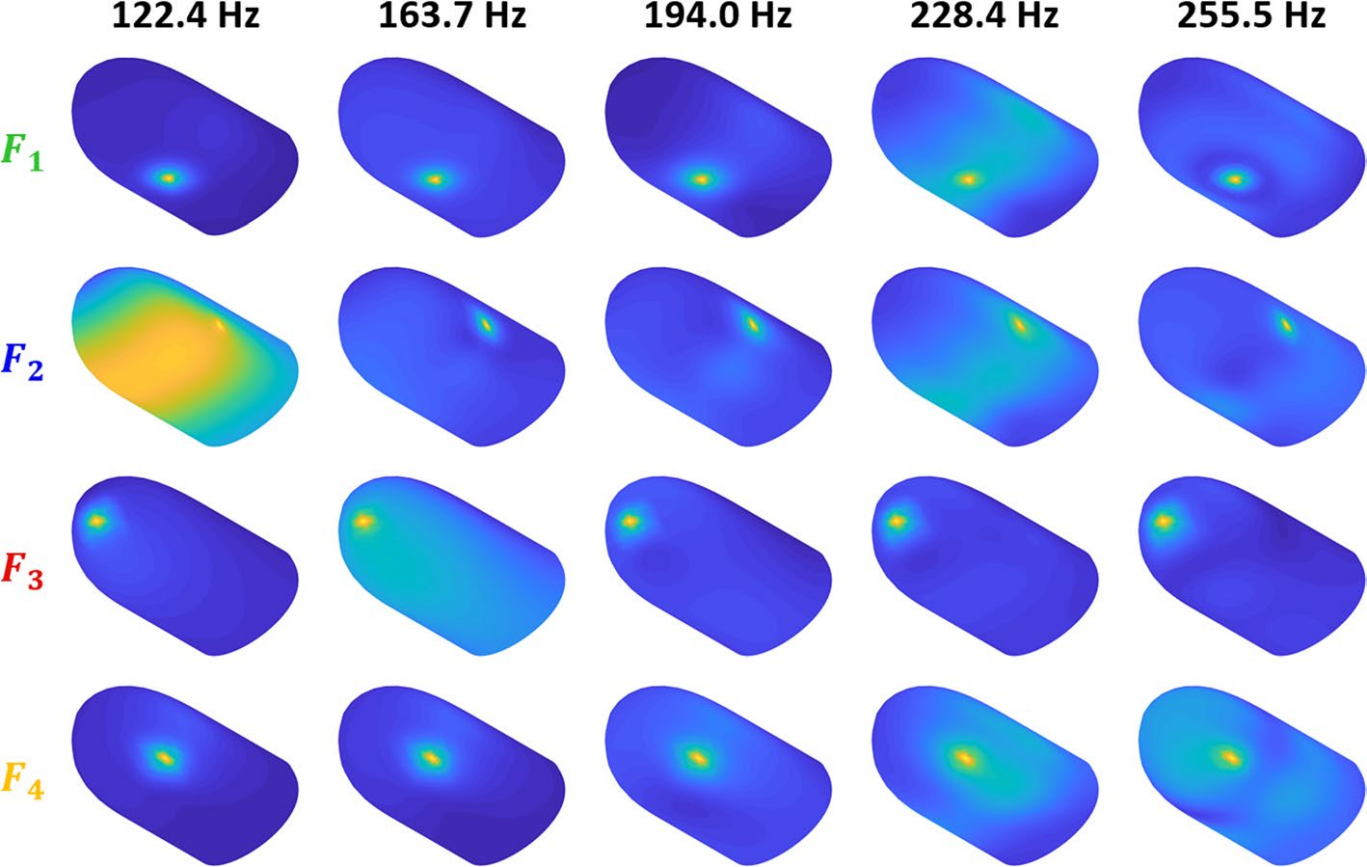
Forced vibration modes around the resonance frequencies for each applied force. Deformation amplitude is depicted with the same color scheme across all twenty plots

For the force applied at the finger pad center ($F_1$), one small peak is observed for the second mode (163.7 Hz), while a significantly stronger one occurs for the fourth mode (225.0 Hz). These results are in accordance with the trends of the displacement FRFs in Fig. 4.

The lateral load $F_2$ strongly excites the first torsional mode (122.4 Hz), resulting in the highest average displacement amplitude across the considered frequency range (Fig. 4). This force also produces a notable peak for the fourth mode due to the coincidence between the excitation point and the modal deformation site.

The force applied at the tip of the finger ($F_3$) produces the strongest resonance for the second mode, while weakly exciting the fourth mode as well. However, this force results in the smallest mean response amplitude when all modes and frequencies are considered. Such low excitation levels can be attributed to the stiffening effects of the relatively thinner skin around the forcing site and nearby harder tissues (i.e., fingernail and nail bed).

The diagonal force $F_4$ causes notable resonances in the responses for all modes from the third to the sixth. Particularly, at frequencies around 240 Hz, significant contributions from the fourth, fifth, and sixth modes are present. This result explains the greater mean displacement amplitudes caused by the oblique excitation at higher frequencies (Fig. 4).

### Forced vibration modes at resonance frequencies

Finally, we compute the forced vibration shapes at the resonance frequencies to investigate the modal factors contributing to the large response amplitudes. For the first three modes, exact resonance frequencies are used to compute the responses. However, the fourth modal frequency (225.0 Hz) is close to the fifth (231.8 Hz), and the sixth modal frequency (254.1 Hz) is close to the seventh (256.8 Hz). For these frequencies, the average values (228.4 and 255.5 Hz) are used since the forced vibration modes for the neighboring frequencies are similar.

Figure 6 shows the results for the four different excitation locations and five selected frequencies. Both parameters have a remarkable influence on the fingertip deformation profile. One can see the correlation between the dispersion of the displacement field and peaks in the mean FRF amplitudes.

In accordance with the mean displacement (Fig. 4, bottom) and modal amplitude FRFs (Fig. 5), the response profiles for $F_2$ at 122.4 Hz and $F_3$ at 163.7 Hz highlight the shapes of the first and second modes, respectively. The deformation is concentrated around the force locations at 194.0 Hz, explaining the absence of significant FRF peaks at this frequency. The shapes at 228.4 Hz reveal that $F_1$ and $F_2$ primarily excite the fourth mode, while the response to $F_4$ mostly reflects the fifth mode. However, all these forces yield comparable mean displacement values at this frequency (Fig. 4, bottom). Hence, similar response amplitudes can be achieved through different resonances when multiple modes are present around the excitation frequency. At 255.5 Hz, the most apparent global motion is observed for $F_4$ due to the collective contribution of the fourth, fifth, and sixth modes (Fig. 5).

### Limitations

The FE analyses that we performed involve inherent limitations. The model was developed using the average characteristics reported for the male index finger; however, fingertip size and shape differ greatly across the population, and tissue properties can vary due to many factors such as hydration (Serhat et al. 2022), age, and gender (Abdouni et al. 2017). The presented results do not reflect the potential influence of any of these parameters on the fingertip’s dynamic response. The FE model is also linear, which limits the validity of the results to small displacements. High excitation amplitudes may induce large deformations that can subsequently produce nonlinear effects. Moreover, we consider only steady-state vibrations and disregard any transient effects (e.g., wave propagation), which have been shown to be important for tactile contacts with the human hand (Shao et al. 2016, 2020).

## Conclusion

This study investigated the dynamic response of the human fingertip to harmonic normal forces with varied locations and frequencies. We utilized a previously developed and validated 3D high-fidelity finite element model to predict the deformation field of the soft tissue. The forces were applied at four representative locations that are commonly used to deliver haptic cues, with stimulation at frequencies up to 260 Hz.

First, we computed the FRFs for the displacement amplitudes at the excitation point and across the entire fingertip. The response curves contain four significant resonances around the first (122.4 Hz), second (163.7 Hz), fourth & fifth (225.0 to 231.8 Hz), and sixth & seventh (254.1 to 256.8 Hz) modes. These FRFs provide clues for choosing force application locations and frequencies to maximize the dynamic response amplitude.

Next, we computed the response amplitudes of individual modes as a function of excitation frequency to investigate the degree of excitation for each mode. The modal displacements explicitly display the contribution of each force to the observed resonances. The results also indicate that larger mean displacement amplitudes can be obtained by applying the excitation in the diagonal direction at the higher frequencies.

Finally, the fingertip vibration patterns associated with each resonance were analyzed. The displacement contours revealed that the excitation point and frequency have significant influences on the dynamic response shapes as well. In addition, we show that similar response amplitudes can be obtained through different resonances in the presence of multiple modes around the excitation frequency. The forced vibration patterns also demonstrate the possibility of controlling the dispersion of vibrations induced by harmonic point forces.

The presented results give insights for selecting the optimal excitation location and frequency to generate fingertip vibrations in scenarios involving dynamic normal forces. One relevant application area is mid-air haptics, which relies on focused ultrasound to create oscillating pressure points on the skin (Hoshi et al. 2010; Long et al. 2014); the vibrations this technology generates are typically noticeable but not strong. Previous research showed that altering the position and modulation frequency of the focal point results in different perceptual effects (Dalsgaard et al. 2022), but the reasons for these variations have not previously been investigated from the perspective of modal dynamics. We envision that our results could be used to target ultrasonic haptic feedback to locations on the finger that will yield more overall fingertip displacement for the same small dynamic input force. The mechanical skin response also depends heavily on frequency due to the demonstrated modal characteristics. Hence, our findings can also be used to predict the frequency at which a small stimulus applied in a particular position on the skin will produce the largest displacements and the strongest sensations. We previously performed such an estimate for a miniature permanent magnet located at center of the finger pad; vibrations driven by the oscillating magnetic field of a nearby air-coil matched well with our experimental measurements (Gertler et al. 2022), but we did not investigate any other magnet locations.

A different scenario where our investigations can be useful is dynamic excitation of fingertips via pressurized air streams, which is another haptic feedback technology (Bianchi et al. 2011; Shultz and Harrison 2022). For air jets having narrow profiles and oscillating amplitudes, the demonstrated excitation mechanisms concerning harmonic point forces could be applicable. The presented vibratory effects could also be beneficial for explaining the frequency-dependent deformation behavior of the fingertip normally excited by pin contactors (Wu et al. 2003).

The model that we utilized was developed for the average male index finger, which is the most commonly reported case in the literature. Future studies should investigate the effects of the model’s parameters on its dynamic response since the fingertip’s characteristics can differ greatly across individuals and environmental factors. Indeed, we believe that such differences may account for a large part of the variability in tactile sensations between individuals: the resonant modes of one’s own fingertips may color what one experiences when feeling any dynamic stimulus. Another promising direction for future work concerns conducting perception experiments. Such investigations can shed light on the relative perceptual importance of deformation amplitudes that occur locally (around the excitation point) versus globally (throughout the fingertip).
